# Methods for capturing and analyzing adaptations: implications for implementation research

**DOI:** 10.1186/s13012-022-01218-3

**Published:** 2022-07-29

**Authors:** Jodi Summers Holtrop, Dennis Gurfinkel, Andrea Nederveld, Phoutdavone Phimphasone-Brady, Patrick Hosokawa, Claude Rubinson, Jeanette A. Waxmonsky, Bethany M. Kwan

**Affiliations:** 1 Department of Family Medicine, University of Colorado, Aurora, CO 80045 USA; 2 Adult and Child Center for Outcomes Research and Delivery Science (ACCORDS), University of Colorado, Aurora, CO USA; 3 Department of Psychiatry, University of Colorado, Aurora, CO USA; 4 University of Houston-Downtown, Houston, TX USA; 5 Department of Emergency Medicine, University of Colorado, Aurora, CO USA

**Keywords:** Implementation fidelity, Adaptation, Shared medical appointments, Qualitative methods

## Abstract

**Background:**

Interventions are often adapted; some adaptations may provoke more favorable outcomes, whereas some may not. A better understanding of the adaptations and their intended goals may elucidate which adaptations produce better outcomes. Improved methods are needed to better capture and characterize the impact of intervention adaptations.

**Methods:**

We used multiple data collection and analytic methods to characterize adaptations made by practices participating in a hybrid effectiveness-implementation study of a complex, multicomponent diabetes intervention. Data collection methods to identify adaptations included interviews, observations, and facilitator sessions resulting in transcripts, templated notes, and field notes. Adaptations gleaned from these sources were reduced and combined; then, their components were cataloged according to the framework for reporting adaptations and modifications to evidence-based interventions (FRAME). Analytic methods to characterize adaptations included a co-occurrence table, statistically based k-means clustering, and a taxonomic analysis.

**Results:**

We found that (1) different data collection methods elicited more overall adaptations, (2) multiple data collection methods provided understanding of the components of and reasons for adaptation, and (3) analytic methods revealed ways that adaptation components cluster together in unique patterns producing adaptation “types.” These types may be useful for understanding how the “who, what, how, and why” of adaptations may fit together and for analyzing with outcome data to determine if the adaptations produce more favorable outcomes rather than by adaptation components individually.

**Conclusion:**

Adaptations were prevalent and discoverable through different methods. Enhancing methods to describe adaptations may better illuminate what works in providing improved intervention fit within context.

**Trial registration:**

This trial is registered on clinicaltrials.gov under Trial number NCT03590041, posted July 18, 2018.

**Supplementary Information:**

The online version contains supplementary material available at 10.1186/s13012-022-01218-3.

Contributions to the literature

- This paper shows how existing methods can be utilized singly and in combination, which is of use to researchers studying translation of evidence-based interventions to practice.
- This paper illustrates, using a case example, specific methods used in combination to highlight ways to understand implementation through examination of adaptations and their descriptive components.
- This paper adds to the literature on methods for studying adaptations, which may be useful for better characterizing which combination of adaptation components is associated with successful implementation outcomes.

## Introduction

A core feature of dissemination and implementation (D&I) research is the conduct of research in a real-world setting with delivery of interventions by existing staff — i.e., pragmatic research [1]. In contrast with efficacy trials, pragmatic D&I studies allow for more flexibility in delivery of the intervention versus requirements to adhere to a strict protocol [1]. As a result, there is expected to be a balance between fidelity to protocol and adaptation to context [2–4]. Fidelity refers to the degree to which an intervention or program is delivered as intended and in accordance with the core elements of the program model [5] and has historically been important in assessing the effectiveness of an intervention in various circumstances based on the extent to which the intervention is delivered in a manner consistent with its most important mechanisms of action. Stirman and colleagues defined adaptations as “changes made to programs or interventions to align them with the context in which they are implemented” [6, 7]. Although adaptations may be deliberately planned to improve fit to context, they can be proactive or reactive [8, 9]. It is important, however, to clearly state a priori those features of an intervention, implementation strategy, or protocol expected to retain fidelity (delivery as intended) — that is, the core elements or “functions” considered essential to effectiveness [10] versus those features more tolerant to adaptation — the peripheral elements that enhance fit to context without diminishing effectiveness.

Given adaptations are to be expected, it is important to systematically track and report on modifications made, including adaptations [2, 11]. By doing so, we can gain understanding on how and why adaptations happen and how they relate to outcomes. Understanding adaptations that occur in real-world contexts can inform both implementation strategy design and selecting interventions that best fit a given context. Reporting on both fidelity and adaptations to suit context and preferences is recommended in the standards for reporting on implementation studies (StaRI) statement [12]. The process of assessing and characterizing adaptations is part of a number of D&I process frameworks, such as the replicating effective programs (REP) framework, and D&I evaluation and planning frameworks, such as RE-AIM [13]. While frameworks and guidance for cataloging adaptations have proliferated in recent years, there are opportunities to expand on how we use and learn from these frameworks.

A key framework examining adaptations in the field of implementation research is the framework for reporting adaptations and modifications to evidence-based interventions (FRAME) [7, 14]. FRAME (and its extension, FRAME-IS, which considers implementation strategies [15]) catalogs adaptation components into “when, how, who, what, and why” descriptors (or “components”) of adaptations. The FRAME further characterizes adaptation components according to context, content, or level of delivery, as well as whether adaptations were planned or unplanned (also called proactive versus reactive) and fidelity consistent or fidelity inconsistent. Using FRAME provides benefit in making adaptations discoverable through their identification and classification. For example, an adaptation might be described as unplanned (planning level), occur during the implementation (when), result from a personnel change (who), because the person terminated their position (contextual, personnel), and for the purposes of reach (i.e., replacing the person, why).

One methodological challenge in the study of adaptations using a framework such as FRAME concerns the data sources and analytic techniques used to characterize adaptations in terms of their descriptive components like the “what, who, when, and why.” Many current approaches commonly involve use of qualitative methods for evaluating implementation outcomes such as fidelity and adaptations [16, 17]. This often utilizes a single data collection method, such as interviews, which may provide a less comprehensive picture of the adaptations. Questions for the field are as follows: what is gained with a multi-method data collection approach, and are there modes of data collection best suited for capturing particular adaptation components and types? Even with multiple data collection methods, analysis can be problematic since adaptations include different descriptive components that can be difficult to interpret. Studying a single component of an adaptation at a time (such as what or who or when or even why) (vs a cluster of adaptation types) may not be instructive in unpacking how adaptations work to improve fit. Examining adaptation components as multidimensional gestalts or types may provide greater insight about how adaptations function, including what components occur together and apart during any one study, and influence outcomes. Thus, another question for the field is as follows: does packaging descriptive components of adaptations together into multidimensional types better characterize them for a future analytical purpose versus examining components individually? A multidimensional cluster approach may be instructive for the field, allowing for a more comprehensive understanding of adaptations. Adaptation types might be more useful for conducting further analyses of adaptations when they become part of an outcomes assessment.

Given these potential methodological questions for the field, this paper expands upon current standards on how the multiple components of adaptations can be effectively captured using multiple data sources and analyzed using FRAME and established techniques from statistical and configurational method approaches. We illustrate how different characterization methods may provide new insights about adaptations from currently available methods. We demonstrate use of these methods to characterize adaptations in the context of a pragmatic comparative effectiveness trial of two models of diabetes shared medical appointments.

## Methods

### Case example: the Invested in Diabetes study

The Invested in Diabetes study is a pragmatic cluster-randomized comparative effectiveness trial designed to compare two models of diabetes shared medical appointments (SMAs) in 22 primary care practices. Practices were randomly assigned to one of two models for delivery of diabetes SMAs: “standardized” or “patient driven.” Practice care team members received training in a diabetes SMA curriculum (Targeted Training in Illness Management; “curriculum”) [18]. Implementation was supported with practice facilitation [19]. Both models involve six diabetes self-management education group sessions using the curriculum; patients also have a visit with a prescribing provider. Patient driven differs from standardized in that patient-driven sessions are delivered by a multidisciplinary care team including health educators, behavioral health providers, and peer mentors (vs a health educator alone in standardized), and patients in patient driven select curriculum topic order and emphasis (vs a set order and prescribed time on each topic in standardized). The enhanced REP framework serves as the D&I process framework guiding the implementation. Enhanced REP includes a “maintenance and evolution phase” in which practice-level fidelity and adaptations to the intervention are tracked. Adaptations during this phase are generally considered reactive and are decided by the local practice implementation teams, as opposed to pre-implementation adaptations, which are planned/proactive and decided upon in partnership by the research and practice teams [4]. The pre-implementation adaptations to the intervention are largely described elsewhere [4, 19].

### Overview

During the REP maintenance and evolution phase of the project — that is, once practices were actively delivering diabetes SMAs — we used several methods to assess fidelity and adaptations. Table 1 outlines the data collection methods and associated instruments, the timing, participants, and the analytic and interpretive process for each of the multiple data sources used to assess fidelity and adaptations. While this paper focuses on methods used to assess and characterize practice-level adaptations made post-implementation, some interview findings reflect pre-implementation adaptations [4] because practice representatives did not necessarily know when the adaptation occurred relative to the REP phases.

**Table 1:**
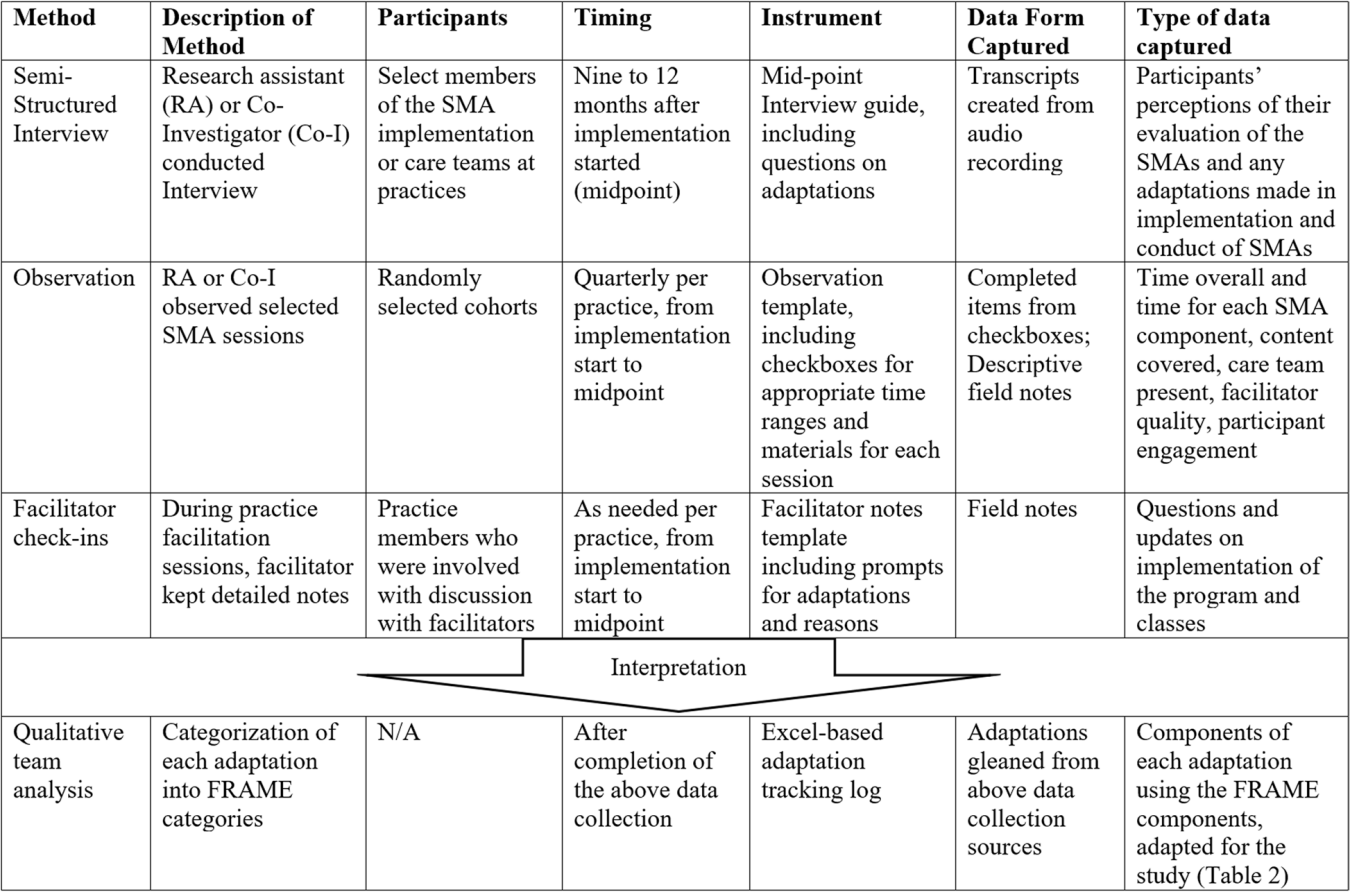
Data collection methods and use for studying adaptations

### Data collection

#### Interviews

Interviews investigated implementation progress and probed specifically for any adaptations made since beginning of implementation. Each individual interview was approximately 60 min, and participants included medical providers, health educators, behavioral health providers, and SMA coordinators. Semi-structured interview guides included specific questions on changes made to either the process of delivering SMAs or the curriculum content delivered during the SMAs. Questions were based on guidance within FRAME and other investigators studying adaptations [14, 20].

#### Fidelity observations

Research staff observed SMA sessions to capture fidelity to both study protocol and curriculum (i.e., personnel used, time of sessions, covered content) as well as elements of the facilitation style and group interaction. The study protocol was to observe one randomly selected session at each participating practice per quarter over six quarters of the study implementation period; the goal was to observe at least one of each of the six curriculum sessions at each practice over the course of the study [19]. This sampling plan was developed in accordance with qualitative data collection standards [21]. The research staff documented fidelity using a structured template which contained checklists to track fidelity of core components of the curriculum and its delivery as well as narrative field notes.

#### Facilitator field notes

The facilitators used templates to document facilitation sessions with the practice site contacts, including implementation challenges and changes made to improve implementation. While the initial four facilitation calls followed pre-planned agendas, additional facilitation sessions that produced written field notes were from ad hoc meetings or emails [4]. The number of facilitation calls (and thus field notes) varied per practice. Field notes were captured in a narrative form in a running shared online platform.

### Data analysis

A core qualitative team analyzed the data. Our team included the qualitative lead (JH), a physician researcher (AN), a postdoctoral fellow (PPB), and a research assistant (DG). All had intimate knowledge of the study protocol and previous qualitative data collection and analysis experience, and all conducted the interviews. A series of steps were conducted to complete the data analysis. These are organized in the Table 2 and explained below.

**Table 2:** Analytic steps and rationale

Step	Reason
1. Primary documents (transcripts, notes) were analyzed; all adaptations found were enumerated.	Allowed us to find all adaptations to the implementation process described
2. Adaptations were entered into a spreadsheet, and each FRAME component was described.	Allowed us to be able to break down and review reasons why adaptations occurred and their intended consequences
3. Adaptations within each practice and data source were de-duplicated.	Quantitizing adaptations allowed us to gather information on how often certain adaptation components occurred and grouped together. Since adaptations were collected through qualitative methods, there was inherent inconsistency in how much any adaptation was identified within data sources. De-duplication removed the issue of conflating number of mentions with number of adaptations as certain interviews could mention the same adaptation multiple times. Keeping de-duplication within each data source allowed us to understand how adaptations occur in each source.
4. Adaptations were compared between data sources.	Allowed us to make recommendations on which types of data collection to use and for what scenarios and intended outcomes
5. Adaptations and their components were enumerated across data sources.	Allowed us to see raw numbers of adaptations/adaptation components discovered in the data
6. Adaptation components were compared using three approaches: co-occurrence, k-means clustering, and taxonomic analysis.	Allowed us to see groupings of adaptations and adaptation components in order to be able to tell an implementation story

#### Identifying adaptations from multiple data sources (step 1)

We utilized a variety of methods to analyze the data. Not all data from all methods (i.e., Table 1) pertained to adaptations and thus were not utilized to capture adaptation information. First, we conducted a traditional qualitative thematic analysis [22] with the interview data. The audio recordings were transcribed into text documents and then uploaded into ATLAS.ti (version 8, Scientific Software Development GmbH). We identified codes using a collaborative process. One of the codes was adaptation, which was defined as any instance of the respondent noting a change from the intended curriculum or process, whether explicitly stated in response to the question — “From when you started, did you make any changes to how you were conducting the sessions or the process?” (e.g., “Yes, we changed the prescribing provider from one of our physicians to the clinical pharmacist”) — or inferred from knowing the protocol and noting that the explanation was different from the intended protocol (e.g., “We utilized our clinical pharmacist as the prescribing provider during the SMAs”). Any changes from the original plan were considered an adaptation; however, they were classified into fidelity consistent or inconsistent as per FRAME and based on the published study protocol [19]. Adaptations were noted into a Microsoft Excel-based adaptations tracking log, described below.

We reviewed each completed observation form and field note from the fidelity observations and facilitator check-ins to identify any adaptations evident in the data. For capturing the adaptations evident in the facilitator and observer notes, we thoroughly reviewed these notes to determine if any changes were made by the practices to the study content/curriculum, processes of implementation at the practice, or elements of the study protocol.

#### Categorizing adaptations using a tracking log (steps 2 and 3)

To organize the data collected from all methods, a tracking log was created in Microsoft Excel structured according to FRAME (Appendix Fig. 1) [2, 11]. This log allowed us to characterize each adaptation by FRAME components (Table 3). Component response choices were taken directly from FRAME; however, some choices were left open-ended or modified to better fit the specifics of the study while keeping to the spirit of FRAME such as “what was modified.” Instead of context, contextual, training and evaluation, or implementation or scale-up activities, we used program content, who was involved, etc. In addition to FRAME components, we added two descriptive categories, “implications of the adaptation” and “what made the adaptation work well/not work well,” so that raters could add narrative comments related to implications and outcomes of the adaptations. Members of the team worked in an iterative process through meetings to refine our common understanding of the terms describing each aspect of FRAME.

**Table 3:** FRAME constructs and additions/clarifications for this study

FRAME element	FRAME component choices (as noted from the model)	Use of FRAME components for this study
*Process*		
When did the modification occur?	• Pre-implementation/planning • Implementation • Scale-up • Maintenance/sustainment	• As our data were primarily from the implementation phase, we did not see data categorized as pre-implementation, scale-up, or maintenance • Split pre-implementation and implementation into “When did it occur” and “For how long did it occur” to distinguish permanence of adaptation
Were adaptations planned?	• Planned/proactive (proactive adaptation) • Planned/reactive (reactive adaptation)	• No modifications
Who participated in the decision to modify?	• Political leaders • Program leaders • Funders • Administrator • Program manager • Intervention developer/purveyor • Researcher • Treatment/intervention team • Individual practitioner • Community members • Recipients	• Changed to reflect relevant roles (researchers, patients, study-involved staff at practice, non-study-involved staff at practice, both researchers and practice staff, other)
What was modified?	• Content • Contextual • Training and evaluation • Implementation and scale-up activities	• Expanded to reflect study changes (program content, who is involved, recruitment, time devoted, follow-up or tracking, scheduling, reimbursement, resources, other)
At what level of delivery (for whom/what is the modification made?)	• Individual • Target intervention group • Cohort of individuals • Individual practitioner • Clinic/unit level • Organization • Network system/community	• Modified slightly to reflect relevant players (individual- patient, individual- practice member, practice, study-initiated for intervention arm, study-initiated for entire project)
Contextual modifications are made to which of the following?	• Format • Setting • Personnel • Population	• Added N/A option
What is the nature of the context modification?	• 15 selection choices including tailoring, packaging, loosening structure, and “drift”	• Answer choices did not fit well with our study, so we did not categorize, opting to capture as part of the open-ended “What was adapted”
Relationship fidelity/core elements?	• Fidelity consistent • Fidelity inconsistent	• As we were comparing two study arms, further expanded “Fidelity inconsistent” to outside protocol (condition specific) for when one study arm was altered to look more similar to the other study arm • Added “Became within protocol” for situations where data reflected an outside of protocol change that was brought back within protocol
*Reasons*		
What was the goal?	• Increase reach or engagement • Increase retention • Improve feasibility • Improve fit with recipient • Address cultural factors • Improve effectiveness/outcomes • Reduce cost • Increase satisfaction	• “Address cultural factors” was merged with “Improve fit with recipient” due to similarities and low number of cultural changes • Added “Outside factors/just happened” and “Other or N/A”
Reasons (sociopolitical, organization/setting, provider, recipient)	• Sociopolitical • Organization/setting • Provider • Recipient	• Not categorized, captured as part of open-ended “Why was it adapted” with free text
*Not in FRAME*		
What we added (not in FRAME)	• N/A	• Free text around implications for time, cost, expertise, etc. (i.e., the impact of the adaptation) • Free text around what made the change go well or not go well

After several rounds of this calibration and cross-checking to achieve a high rate of consistency in scoring across the research team, we divided up the data for each practice and completed the tracking log until all adaptations found in all forms of data collection (interviews, observation templates, and facilitator notes) were tracked for each practice. Then each adaptation was reviewed and de-duplicated by practice (explained in the Table 2) and data source by DG, reducing the data set to include one description for each unique adaptation found by data source for each practice. The resulting document included each adaptation with descriptive information. Other team members (AN and PPB) reviewed the document for accuracy.

#### Comparing the data sources (step 4)

We created a second spreadsheet to determine the concordance/discordance (similar to agreement/disagreement) of the adaptation information revealed by each data source. Using our modified categories around “What was adapted” from FRAME (follow-up or tracking, program content, recruitment, resources, scheduling, time devoted, who is involved, and other), we completed a table noting areas of adaptation that were found in each data source and then scored (from 1 to 4) the degree to which the data found in each source was the same, somewhat the same, or different. Data was scored by all team members and reviewed at team meetings. Once consensus was achieved on scoring, a single reviewer (DG) finished rating all adaptation differences. Any uncertainty was brought up to the full team to review. The scores were then summarized across all practices (mean, count), and we wrote a summary of how data in each category differed or converged across the data sources. This produced a summary of total adaptations and concordance across the data sources and is represented in Table 4 in the “Results” section below.

**Table 4:** Concordance/discordance of adaptations found across data sources

Adaptation domain — what was adapted (FRAME)	Degree of concordance/discordance across data sources	Summary of reasons for disparities
Follow-up or tracking (Ex: began contacting patients before sessions)	Mean score: 2.3 Counts Completely different (1): 8 Some similarities (2): 2 Mostly similar (3): 6 Same data (4): 4	• Interview and facilitator note data was similar in sharing stories of changes to participant follow-up and data collection • The most descriptive data was in the facilitator notes with regard to understanding and asking for permission on patient-reported outcomes • This was generally not covered in the observations unless a survey was to be completed during the specific class session in which it could be seen if this was done or not
Program content (Ex: not all curriculum content covered during session)	Mean score: 2.2 Counts Completely different (1): 4 Some similarities (2): 10 Mostly similar (3): 4 Same data (4): 2	• Found in all data sources but different emphasis and perspectives • The interview data provided more perceptions of the curriculum and why changes were made, the facilitator notes reported difficulties with the content and the observations noted when content had been altered but not why
Recruitment (Ex: expanded focus of recruitment past patients with high A1c)	Mean score: 2.4 Counts Completely different (1): 7 Some similarities (2): 2 Mostly similar (3): 7 Same data (4): 4	• Almost all practices had adaptations in their recruitment strategies, ranging from small changes to completely different strategies. This was discussed in interviews and in facilitator notes, with differences in levels of detail • Almost entirely missing from observations, except one key point (type 1 diabetic patient found)
Resources (Ex: began utilizing whiteboard)	Mean score: 2.6 Counts Completely different (1): 9 Some similarities (2): 1 Mostly similar (3): 0 Same data (4): 10	• Mostly, no data reported in any source (4), but when it was reported, tended to come out in either interviews or facilitator notes or both • Observations were lacking in this data
Scheduling (Ex: changed to weekly sessions from monthly sessions)	Mean score: 2.1 Counts Completely different (1): 9 Some similarities (2): 2 Mostly similar (3): 7 Same data (4): 2	• Mostly reported in interviews, sometimes in facilitator notes • Not reported in observations
Time devoted (Ex: classes shorter than 120 min)	Mean score: 1.7 Counts Completely different (1): 13 Some similarities (2): 2 Mostly similar (3): 3 Same data (4): 2	• Primarily mentioned only in observations, coming across as shorter sessions • Other sources sometimes showed differences in time devoted by administrative personnel
Who is involved (Ex: class facilitator resigned and replaced)	Mean score: 2.8 Counts Completely different (1): 2 Some similarities (2): 4 Mostly similar (3): 11 Same data (4): 3	• Some similarities between interviews and facilitator notes • Seemed to come in to play from all sources
Other (Ex: practice staff began using instant messaging rather than meetings)	Mean score: 2.9 Counts Completely different (1): 6 Some similarities (2): 2 Mostly similar (3): 0 Same data (4): 4	• Mostly not reported by any source, likely due to good characterization of data • Mostly came from one source for each practice, varied between observations and interviews • Did not occur in observations
Overall	Mean score: 2.4 Counts Completely different (1): 58 Some similarities (2): 25 Mostly similar (3): 38 Same data (4): 39	• All data sources had unique data present • Observation data was most relevant for timing of sessions • Facilitator note data had most instances of background for adaptations • Interviews and facilitator notes matched up a lot of the time; observation data was more likely to be independent • 36% of data was completely different between sources

Data is from 21 practices, condensed to 20 due to coupling of data for one practice group. Mean score of rating 1–4. Each domain was compared 20 times

#### Identifying the adaptation components and clusters (steps 5 and 6)

In order to understand what adaptation components were observed, we summarized each FRAME component of each adaptation separately and reviewed how many times each component was distributed across the data sources. Next, we used three analytic approaches to categorize adaptation components into meaningful types. First, we created a co-occurrence table (or cross tabs), to assess the frequency with which each component coexisted with one other component in a 2 × 2 table. Table 5 shows the co-occurrence for type of adaptation (process/implementation versus content/sessions) compared with all other adaptation components (who, what, when, etc.). Other configurations of adaptation components could be considered as needed.

**Table 5:** Data captured in the adaptations tracking log

		Adaptation type (FRAME)		
		Process/implementation	Classes/program	Total
FRAME adaptation descriptor	Process/implementation	123	0 (N/A)	123
	Classes/program	0 (N/A)	79	79
What was adapted	Follow-up or tracking	21	1	22
	Program content	0	49	49
	Recruitment	43	1	44
	Resources	3	5	8
	Scheduling	17	3	20
	Time devoted	7	8	15
	Who is involved	26	12	38
	Other	6	0	6
Why was it adapted	Increase reach/engagement	36	5	41
	Increase retention	5	1	6
	Improve feasibility	41	8	49
	Improve fit with recipients	2	13	15
	Improve outcomes	6	21	27
	Reduce cost	6	1	7
	Increase satisfaction	7	10	17
	Other or N/A	6	12	18
	Outside forces/ “just happened”	14	8	22
Adaptation level (planning)	Planned (proactive)	13	18	31
	Unplanned (reactive)	110	61	171
Adaptation level (fidelity)	Within protocol	116	46	162
	Outside of protocol	5	24	29
	Outside of protocol (condition specific)	0	5	5
	Became within protocol	2	4	6
Delivery level	Individual-patient	2	0	2
	Individual-practice member	21	38	59
	Practice	97	41	138
	Intervention group	0	0	0
	All practices	0	0	0
Context modifier	Format	8	46	54
	Setting	6	1	7
	Personnel	38	12	50
	Population	25	1	26
	N/A	46	19	65
When did it occur	Proactive — planning	11	9	20
	Reactive — during or after 1st class	43	30	73
	Reactive — later into implementation (after 2nd)	29	9	38
	Unclear	40	31	71
For how long did it occur	Temporary adaptation	2	8	10
	Permanent adaptation	103	50	153
	Evolving adaptation	5	1	6
	Unclear	13	20	33

Second, we used statistically based k-means clustering to identify patterns in the adaptation data by grouping together adaptation components into a pre-specified “k” number of clusters, resulting in groups of adaptations that had similar adaptation constructs which could be reviewed and interpreted by study team [23, 24]. The resulting clusters helped describe patterns for how adaptation components fit together. At first, only the elements of FRAME deemed most critical were included— the adaptation type, why was it adapted, when was it adapted, the planning level, and level of fidelity — which capture the adaptation as well as crucial contextual factors (e.g., when and why it occurred). Then other components were included to see if the additions produced insight to the results — i.e., did new groups created seem to go together well. In Tables 6 and 7, we present clusters with five and seven components identified qualitatively as most important to include with clusters staying more or less homogeneous. Other choices not selected for inclusion in the clustering were deemed less likely to influence eventual outcomes (e.g., for how long it occurred, delivery level).

**Table 6:**
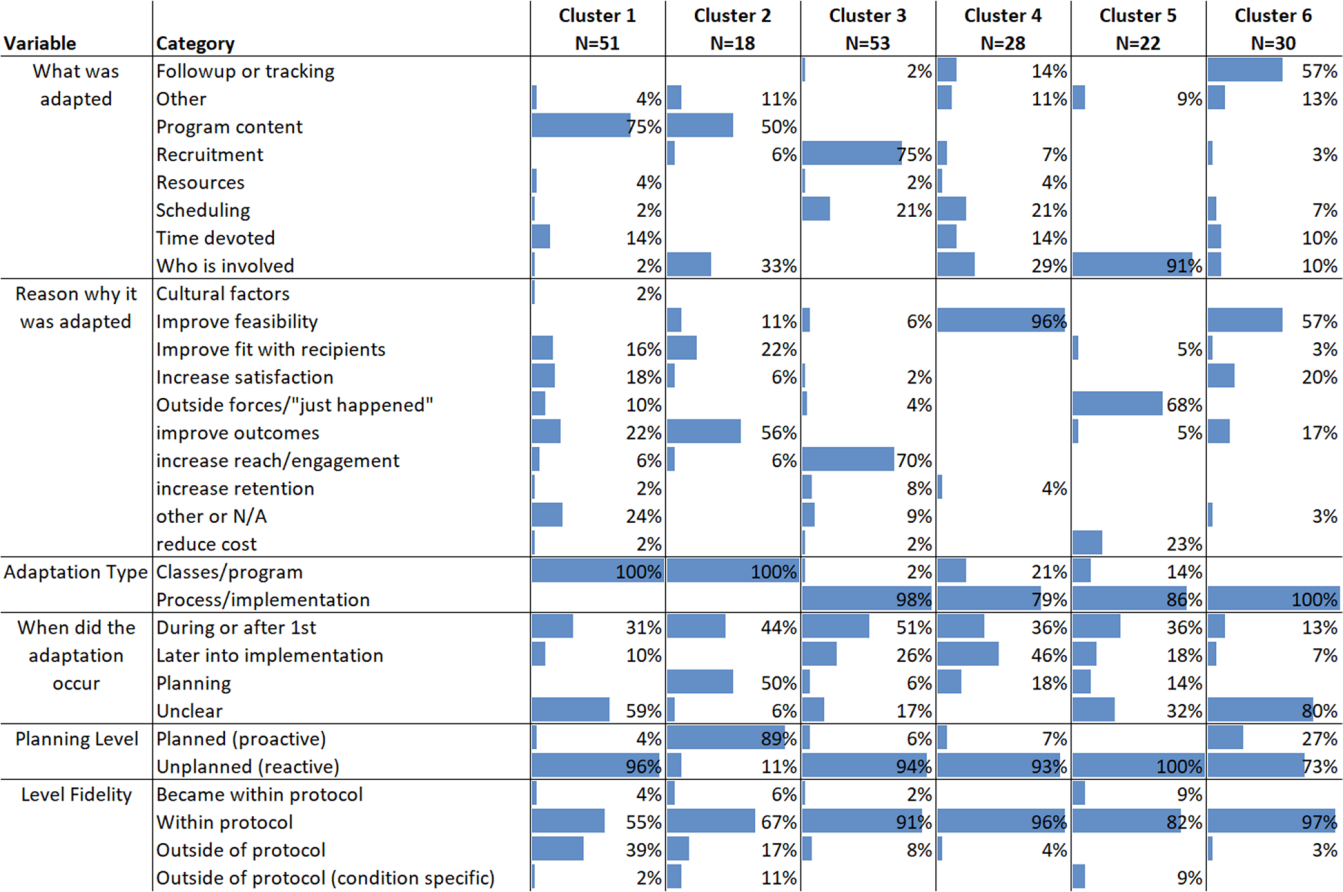
Five adaptation components cluster model. Clusters shown in Table 6 can be roughly summarized as the following: Cluster 1: unplanned program content changes for a variety of reasons that could go against study protocol. Cluster 2: Planned program content changes early on to improve outcomes. Cluster 3: Unplanned changes to practice processes (recruitment and scheduling) early on to improve reach/engagement. Cluster 4: Unplanned implementation changes of various sorts for the reason of improving feasibility that happened throughout implementation. Cluster 5: Unplanned reactionary changes throughout the implementation to study personnel. Cluster 6: Unplanned changes to a variety of process areas with the goal of improving feasibility through the implementation process

**Table 7:**
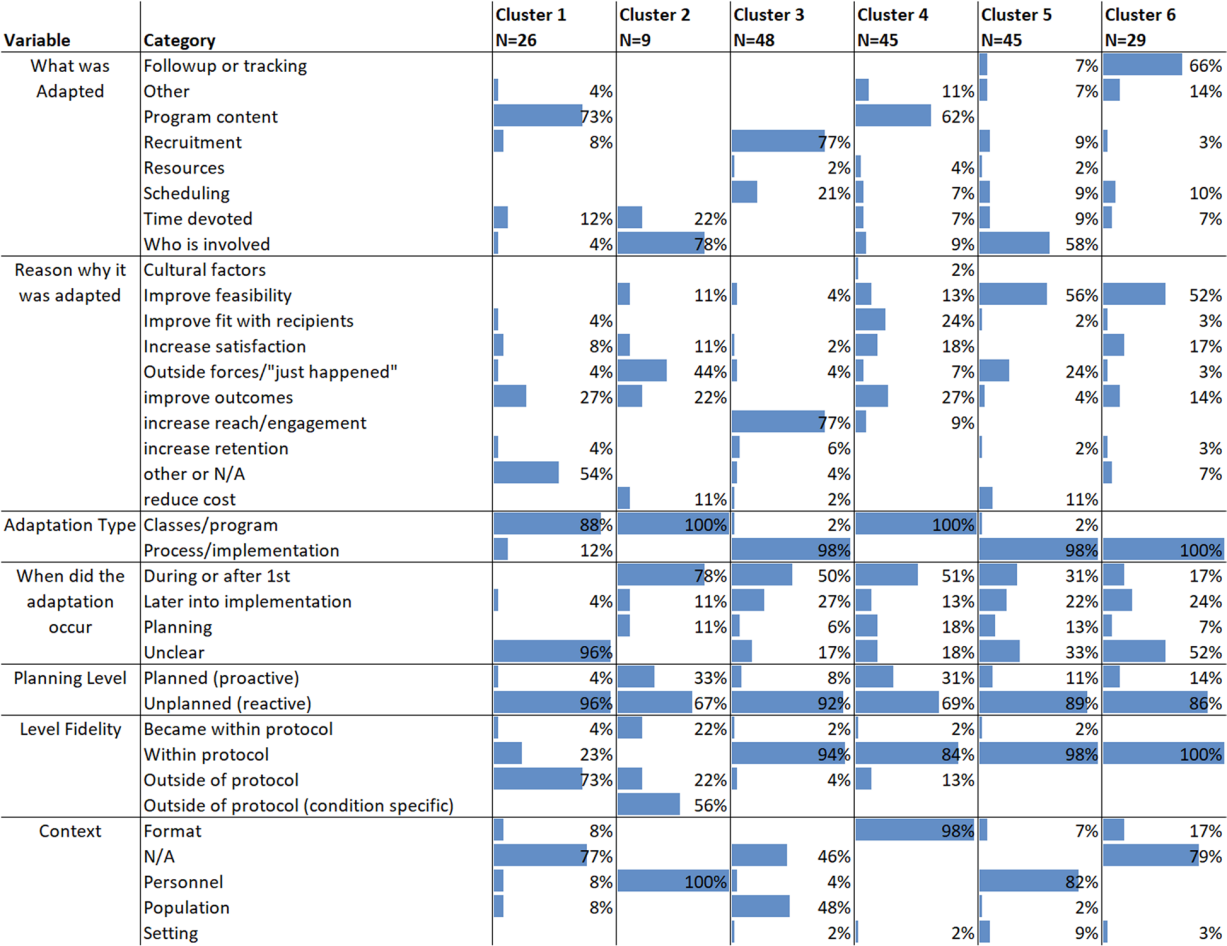
Seven adaptation components cluster model. Clusters shown in Table 6 can be roughly summarized as the following: Cluster 1: Unplanned program content changes for a variety of reasons that were outside protocols at unknown times and were mostly outside of study protocol. Cluster 2: Unplanned changes to study personnel early on that involved who was involved and were mostly outside of study protocol. Cluster 3: Unplanned changes to practice processes (recruitment and scheduling) early on to improve reach/engagement. Cluster 4: Unplanned program content changes for a variety of reasons at a variety of times that were within protocol. Cluster 5: Unplanned reactionary changes throughout the implementation to improve feasibility that largely affected who was involved. Cluster 6: Unplanned changes to follow-up or tracking with the goal of improving feasibility through the implementation process

Finally, we performed a taxonomic analysis, a configurational-comparative technique that identifies all possible different combinations of components and how they interact. Adaptation component combinations were added into a table identifying all possibilities present in the data [25]. To produce a table that was a reasonable size to interpret, we narrowed the number of included adaptation components, consistent with those in k-means cluster Table 6 so that comparisons could be made across methods. We separated the analysis by process/implementation and sessions/content, allowing us to identify patterns of how components of adaptations clustered together (i.e., how types of adaptation components paired with others) from different perspectives. The resulting tables showed each possible combination of adaptation components present in the data, along with the count of how often they occurred. These results are displayed in Tables 8 and 9.

**Table 8:** Adaptations within classes/content

Why did the adaptation occur?							
What was adapted?	Fidelity	Improve feasibility (During planning: after planning)	Increase reach/engagement (During planning: after planning)	Increase satisfaction (During planning: after planning)	Improve outcomes (During planning: after planning)	Improve fit (During planning: after planning)	Other/NA (During planning: after planning)
Content (*n* = 47)	Consistent (*n* = 31)	0:0	0:3	0:4	2:10	2:6	0:4
	Inconsistent (*n* = 16)	0:0	0:1	0:2	1:2	0:2	0:8
Who is involved (*n* = 12)	Consistent (*n* = 5)	1:2	0:0	0:0	0:0	1:0	0:1
	Inconsistent (*n* = 7)	0:1	0:0	0:1	1:2	0:0	0:2
Time devoted (*n* = 8)	Consistent (*n* = 2)	0:1	0:0	0:1	0:0	0:0	0:0
	Inconsistent (*n* = 6)	0:1	0:0	0:1	0:0	0:0	0:4
Scheduling (*n* = 3)	Consistent (*n* = 3)	0:1	0:1	0:1	0:0	0:0	0:0
	Inconsistent (*n* = 0)	0:0	0:0	0:0	0:0	0:0	0:0
Resources (*n* = 2)	Consistent (*n* = 2)	0:0	0:0	0:0	0:1	0:0	0:1
	Inconsistent (*n* = 0)	0:0	0:0	0:0	0:0	0:0	0:0
Follow-up (*n* = 1)	Consistent (*n* = 1)	0:1	0:0	0:0	0:0	0:0	0:0
	Inconsistent (*n* = 0)	0:0	0:0	0:0	0:0	0:0	0:0
Recruitment (*n* = 1)	Consistent (*n* = 1)	0:0	0:1	0:0	0:0	0:0	0:0
	Inconsistent (*n* = 0)	0:0	0:0	0:0	0:0	0:0	0:0
Other/NA (*n* = 5)	Consistent (*n* = 5)	0:1	0:0	0:0	1:1	0:2	0:0
	Inconsistent (*n* = 0)	0:0	0:0	0:0	0:0	0:0	0:0

**Table 9:** Adaptations within process/implementation

Why did the adaptation occur?							
What was adapted?	Fidelity	Improve feasibility (during planning: after planning)	Increase reach/engagement (during planning: after planning)	Increase satisfaction (during planning: after planning)	Improve outcomes (during planning: after planning)	Improve fit (during planning: after planning)	Other/NA (during planning: after planning)
Recruitment (*n* = 43)	Consistent (*n* = 39)	0:6	1:27	0:1	0:0	0:0	2:2
	Inconsistent (*n* = 4)	0:0	0:2	0:0	0:0	0:0	0:2
Who is involved (*n* = 26)	Consistent (*n* = 26)	2:12	0:0	0:0	1:0	0:1	1:9
	Inconsistent (*n* = 0)	0:0	0:0	0:0	0:0	0:0	0:0
Follow-up (*n* = 21)	Consistent (*n* = 21)	0:10	0:0	0:5	0:5	0:0	0:1
	Inconsistent (*n* = 0)	0:0	0:0	0:0	0:0	0:0	0:0
Scheduling (*n* = 16)	Consistent (*n* = 16)	1:5	0:10	0:0	0:0	0:0	0:0
	Inconsistent (*n* = 0)	0:0	0:0	0:0	0:0	0:0	0:0
Time devoted (*n* = 6)	Consistent (*n* = 6)	1:5	0:0	0:0	0:0	0:0	0:0
	Inconsistent (*n* = 0)	0:0	0:0	0:0	0:0	0:0	0:0
Resources (*n* = 2)	Consistent (*n* = 2)	0:1	0:1	0:0	0:0	0:0	0:0
	Inconsistent (*n* = 0)	0:0	0:0	0:0	0:0	0:0	0:0
Content (*n* = 0) (*n* = 1)	Consistent (*n* = 0)	0:0	0:0	0:0	0:0	0:0	0:0
	Inconsistent (*n* = 0)	0:0	0:0	0:0	0:0	0:0	0:0
Other/NA (*n* = 9)	Consistent (*n* = 8)	1:3	0:0	0:0	0:0	0:1	1:2
	Inconsistent (*n* = 1)	0:0	0:0	0:1	0:0	0:0	0:0

## Results

This analysis included 72 practice member interview transcripts, 33 completed observation forms, and 168 facilitator field notes, a total of 273 documents representing data collection through the midpoint of the implementation period. Our key findings are summarized below.

### Finding no. 1: different methods elicited more overall adaptations

Table 4 reports the concordance/discordance of the adaptations revealed from each of the three data sources, broken down by the “What was adapted” construct. Given that the scores tended to be near 2 out of 4 instead of 4 out of 4, this demonstrates the importance of using all three data sources to fully evaluate adaptations, as this approach made it more likely that an adaptation was identified. Observation data were the best source of information about what changes had been made to the curriculum, who was filling which roles in delivering SMAs, and how long sessions lasted. This information was often absent from the interview transcripts or facilitator notes. Thus, without observation data, information about those adaptations would not have been revealed. Conversely, session observations revealed no information about why any adaptations took place, for which the facilitation notes or interview transcripts were much more insightful. Using only observations to evaluate adaptations would have left questions about why the adaptations were made and if they were intentional or unintentional. The facilitator field notes were perhaps the best source of information on major process changes because difficulties with process elements were often discussed with facilitators. Interview data may have been prone to recall bias because interviews were often conducted much later than the adaptation occurred and may have varied by the particular participant interviewed. Due to staff turnover, some interviewees did not have the background knowledge necessary to comment on change in process over time. However, specific questions and probes focused on adaptations were asked in interviews, so knowledgeable respondents could discuss many adaptations and reasons for them in a short amount of time. In summary, different types of data collection appeared to be inherently better for capturing different types of information. Overall, data were somewhat to mostly similar across all sources (mean score on concordance/discordance of data across all sources was 2.4 out of a scale of 1–4).

### Finding no. 2: different data collection methods provided greater understanding of the components of and reasons for adaptation

Across the 21 practices, there were a total of 202 adaptations when all duplicates within data sources were removed (Table 5). All practices reported at least three unique adaptations discovered from any method (range 3–22; mean of 9.6). As shown in Table 5, more adaptations occurred in process/implementation (*n* = 123) than in the sessions/program (*n* = 79), with most of the adaptations across both types being unplanned/reactive (171) with the goal of improving the feasibility (49), reach (41), or outcome (27). It is also important to note that the overwhelming majority were within the intervention protocol (162) noting that the fidelity to the core elements of the intervention was high, and that most of what was being captured were changes to improve the fit of the intervention to the contextual circumstances rather than to change the intervention.

### Finding no. 3: different analytic methods revealed ways that adaptation components cluster together in unique patterns, producing adaptation “types”

Beyond listing the adaptation components, there was interest in determining whether and how these components clustered together.

First, Table 5 shows how the different components associated with the adaptation type (process/implementation vs. sessions/program). The associations have high face validity. For example, program content was always in the adaptation type of sessions/program (49/49), whereas follow-up/tracking and recruitment were almost always in the adaptation type of process/implementation (21/22 and 43/44, respectively). Other components of adaptation were split more evenly between the adaptation types.

To go beyond analysis of adaptation characteristic pairings, Tables 6 and 7 demonstrate the output from two iterations of the k-means cluster analyses. Table 6 demonstrates when the analysis was conducted with five adaptation components, whereas Table 7 includes seven. We found that groupings of components held fairly stable within clusters (though were not identical) regardless of how many elements were added. Looking at the columns of both tables reveals how many components tend to cluster. For example, Table 6 describes a cluster around program content adaptations that were done primarily to improve outcomes and other reasons, and another where adaptations were primarily done to improve fit or increase satisfaction. Each cluster in Tables 6 and 7 showcases adaptation cluster types, which could be used to describe what types of adaptations are occurring and why. In Table 6, clusters 1 and 2 both include adaptations to the program content (what was adapted) which correspond with the classes/program (adaptation type). However, they differ in that cluster 1 was unplanned (planning level) and had some both within and not within protocol, whereas cluster 2 was planned and more typically within protocol. Clusters 3, 4, and 5 all related to process/implementation (adaptation type) but differ with what adapted and the reason it was adapted. They all were largely unplanned. Some of these cluster types were more uniform in the reported elements, such as cluster 3 in Table 6. Other types had one element held constant but others more diversified, such as cluster 1 in Table 6, which seems to show an adaptation archetype around program content, which occurs for a variety of reasons. It is instructive to note that when an adaptation was outside of protocol, it was most likely to occur in the package (i.e., cluster 1) that was about program content, was intended to improve outcomes, was not clear when it occurred, and was unplanned. Thus, adaptations within protocol were much more often process in nature, which perhaps reflected the more natural ability of processes than curricular items to be adapted without fidelity violations. Adaptation clusters are further described in Tables 6 and 7.

In contrast, the configurational taxonomic analysis (Tables 8 and 9) shows each independent combination of the adaptation components. Out of 414 possible iterations of adaptation component configurations, only 69 actually occurred. Both Tables 8 and 9 use the same five components, but we display them as two tables by the adaptation type (8 showing sessions/content adaptations vs 9 showing process/implementation adaptations) to aid in interpretation. Adaptations to the sessions/content showed overwhelmingly “content” as the most commonly adapted element (*n* = 47, 59%), and when that happened, there were fidelity consistent (*n* = 31) and inconsistent (*n* = 16) adaptations which most often occurred to improve outcomes (*n* = 12) or improve fit (*n* = 8). The “what” that occurred within process/implementation were more evenly distributed, most often recruitment (*n* = 43, 35%), who was involved (*n* = 26, 21%), or follow-up (*n* = 21, 17%). The reason for adaptation varied more in the sessions/content category but was primarily driven by improving feasibility (*n* = 47, 38%) and increasing reach/engagement (*n* = 41, 33%). The “when adapted” varied a lot in combination with all the other combinations in the sessions/content category but was primarily after planning for the process/implementation category.

## Discussion

In these analyses, we found FRAME to be useful for illustrating the adaptations practices made while implementing diabetes SMAs in the context of a pragmatic effectiveness-implementation trial with multiple forms of data collection to assess fidelity to protocol. One benefit of comparing different sets of data collection methods was greater coverage of all the adaptations made. Not only were multiple methods beneficial in finding more adaptations, the different methods uncovered different types of adaptations, providing a more comprehensive view of adaptations. Using only one data collection method may create gaps in uncovering all of the adaptations as well as the how and why of the adaptations. Existing literature on adaptations characterized using single methods may therefore be missing certain types of adaptations. Indeed, McCarthy et al. found that three different data collection methods yield nonoverlapping adaptation data, i.e., none of the adaptations was found in all three data collection methods. They further explain that the multiple methods allowed for triangulation and richer understanding [26]. We found much more overlap across adaptations by source than McCarthy reports but still validate the benefit of multiple data collection methods providing richer understanding of the adaptations. On the other hand, a multi-method approach to assessing adaptations can be time-consuming and costly, and the cost–benefit of this approach may not be worthwhile for all projects. Because we also found that different data collection methods provided greater understanding of the components of and reasons for adaptation, a potential recommendation from finding no. 1 results might be a form of interview or discussion-based data collection method (such as structured facilitator field notes) with some form of observation to capture what is happening in the actual intervention as well as allowing participants to explain their choice of changes.

Another finding was that different analytic methods revealed ways that adaptation components cluster together in unique patterns providing adaptation “types.” When we looked at the different adaptation components, creating a simple co-occurrence (using cross-tabs) was useful in understanding the complexity of the adaptations (i.e., looking at what adaptation components occurred and why they occurred together); however, we also gained additional insight from the cluster and configurational analyses. From an academic or methods perspective, this approach helped to easily identify what combinations of components are present in adaptations observed. The k-means cluster method allowed us to see a gestalt of the adaptation data, while the configurational approach allowed more granularity than the simple cross-tab in the co-occurrence table. It also allowed us to easily discern patterns in data (e.g., if fidelity is consistent, what happened, and why?) and spot more one-off adaptations (i.e., if the identified adaptation always occurs in a certain pattern or if the particular observation is an anomaly). For example, fidelity-consistent adaptations around content which were done to improve outcomes were relatively frequent, occurring 12 times, while fidelity-inconsistent adaptations around content done to increase reach were not frequent, occurring only once. This may become more useful with larger datasets, as we observed a lot of singular adaptation configurations; however, more data could also bring in more configurations, making the table larger and harder to interpret. Knowing this may have implications for making sense of the need for particular course-correcting implementation strategies. For both methods, we decided to group these variables together in this way to fit our dataset, but this is highly customizable. The data could be displayed differently, or different components could be selected, which makes these methods versatile. From an implementation point of view, the configurational format in particular allows implementers to locate what is being adapted, why, when, and how in a way that is easy to quantify and observe, allowing for decision-makers to adjust the protocol or understand why implementation is straying from fidelity. The versatility again makes it useful, because it aligns the data with the desired research question or purpose. The k-means clustering approach, on the other hand, can be useful to determine your top adaptation combinations, which could also be useful in planning fidelity maintenance.

This paper illustrates the potential benefit of having more data sources about adaptations and use of a framework like FRAME to catalog adaptation components and to subsequently cluster the components together for more descriptive insight. However, describing the components of adaptations is only one step in the process. A next logical step could be to consider how clusters of adaptation types correlate with outcomes, such as with the model for adaptation design and impact (MADI) [27] After having characterized adaptations (such as one might do using FRAME or FRAME-IS, which are a part of MADI), one might examine effects of different types of adaptations on intervention and implementation outcomes. For instance, adaptations to program content (e.g., adding a module to an educational intervention) or level of delivery (e.g., delivering an intervention to both a patient and a care partner versus a patient alone) may yield improved patient clinical outcomes or may make the intervention more acceptable in a particular setting.

There are several limitations of this work. Although the focus of this paper is on the methods conducted, the results of the adaptations found are also included to illustrate our methodological points. Therefore, caution should be taken in interpreting the tabular results in that these data were provided from qualitative information gathered from practices in two states participating in a study of implementing diabetes SMAs which may not represent other practices in other geographic areas and circumstances. All interview candidates were asked the same questions about adaptations; however, specific types of responses were not systematically requested. Therefore, it is possible that not all responses for all participants were recorded, and when summed, this may have misrepresented the number of adaptations made. As mentioned, interviews may have suffered from recall bias. Also, some attrition in staffing may have made some adaptations not reportable. Additionally, when completing observations, our observers may have misinterpreted observed information, and since they were not able to ask, we had to infer or select “other” or “unknown” for the explanation as to the why for those adaptations that were not reported in our other data collection methods. Also, this analysis utilizes quantitizing (converting qualitative data into quantitative data), which may have resulted in not accurately capturing the intended meaning in the conversion from words to numbers. Different methods also produced different adaptations through time such that interviews were at a point in time and asked respondents to look back over time, whereas each observation occurred only at one point in time and field notes occurred over time. In conducting these types of analyses, we limited adaptation components to one per category (e.g., the reason for adaptation was to improve fit or outcomes or reach/engagement but not all three) as to highlight the most important aspect of each adaptation rather than capture all aspects of an adaptation characteristic. Therefore, we might have not selected the most important adaptation characteristic or represented them in overly simplistic ways. Last, we chose to utilize the FRAME to categorize our responses generally but did intentionally make some slight clarifications and additions to it to fit our study. These modifications may add to the findings from our study but may make comparisons across other projects utilizing FRAME challenging. Additionally, FRAME-IS has recently emerged as a further framework that might have fit our project better but was not available at the time of this analysis; we also did not analyze adaptations to the implementation strategies, which is the focus of FRAME-IS. Finally, since data was part of a midpoint analysis, and did not include pre-implementation data, we mostly captured data around implementation adaptations.

## Conclusion

In summary, adaptations and how to characterize them are important to pragmatic research and D&I science. We found that adaptations were prevalent and made more discoverable through different methods. Characterizing the adaptations into description units (clusters or tables) helped to illuminate how the adaptation components tended to associate together (or not), which may be useful to associate with eventual program outcomes. This paper is meant to be a foundation for methods and ways of examining adaptations and the interplay between different adaptation components. We recommend that researchers and program implementers use multiple methods to capture adaptations and consider how they may package them into analyzable units such as produced by clustering or co-occurrence for enhanced understanding of the phenomena they are studying. This may help facilitate use of MADI and other analysis in tying adaptation components to outcomes. In the end, having a better sense of what works will be helpful in driving program efforts for implementation in clinical and public health settings.

## Supplementary Information

**Additional file 1.** FRAME.

## Data Availability

The datasets analyzed during the current study are available from the corresponding author on reasonable request.

## Abbreviations

D&I: Dissemination and implementation
StaRI: Standards for reporting on implementation studies
REP: Replicating effective programs
FRAME: Framework for reporting adaptations and modifications to evidence-based interventions
MADI: Model for adaptation design and impact
SMAs: Shared medical appointments
